# Intake of Dairy Products, Calcium, Magnesium, and Phosphorus in Childhood and Age at Menarche in the Tehran Lipid and Glucose Study

**DOI:** 10.1371/journal.pone.0057696

**Published:** 2013-02-25

**Authors:** Fahimeh Ramezani Tehrani, Nazanin Moslehi, Golaleh Asghari, Roya Gholami, Parvin Mirmiran, Fereidoun Azizi

**Affiliations:** 1 Reproductive Endocrinology Research Center, Research Institute for Endocrine Sciences, Shahid Beheshti University of Medical Sciences, Tehran, Iran; 2 Obesity Research Center, Research Institute for Endocrine Sciences, Shahid Beheshti University of Medical Sciences, Tehran, Iran; 3 Department of Clinical Nutrition and Dietetics, Faculty of Nutrition Sciences and Food Technology, National Nutrition and Food Technology Research Institute, Shahid Beheshti University of Medical Sciences, Tehran, Iran; 4 Endocrine Research Center, Research Institute for Endocrine Sciences, Shahid Beheshti University of Medical Sciences, Tehran, Iran; University of Washington, United States of America

## Abstract

**Purpose:**

Studies indicate that milk intake is associated with insulin-like growth factor 1 (IGF-1) concentrations and height in childhood, whether milk and other dairy products promote puberty remains unclear. This study aimed to investigate influences of pre-pubertal intakes of milk, yogurt and cheese on menarcheal age in Tehranian girls. The associations of total dietary calcium (Ca), magnesium (Mg), and phosphorus (P) with menarcheal age were also examined.

**Methods:**

This prospective study was conducted on 134 pre-pubertal girls, aged 4-12 years at baseline, who participated in the Tehran Lipid and Glucose Study (TLGS), and were followed for a median of 6.5 years. Dietary intakes were determined at initiation of the study using two non-consecutive 24-h dietary recalls and the age of menarche was documented during the follow-up. Logistic regression was used to calculate the risk of reaching menarche ≤ 12 years according to pre-pubertal levels of dairy or mineral intakes.

**Results:**

The risk of earlier menarche was higher in girls with higher intakes of milk [OR: 2.28 (95% CI: 1.03–5.05)], Ca [OR: 3.20 (95%CI: 1.39–7.42)], Mg [OR: 2.43 (95% CI: 1.12–5.27)] and P [OR: 3.37 (95 % CI: 1.44–7.87) after controlling for energy and protein intake, interval between the age at study initiation and the age of menarche, and maternal age at menarche (Model 1). Girls in the middle tertile of cheese intakes had a lower risk of reaching menarche ≤ 12 years than those in the lowest tertile after controlling for covariates in model 1. These associations remained significant after further adjustment of BMI Z-score at baseline. The relationship of Ca, Mg, and P with menarche remained after further adjustment for height Z-score at baseline, whereas the association between milk and cheese intakes became non-significant.

**Conclusions:**

Pre-pubertal intake of milk, but not cheese and yogurt, may hasten age at menarche.

## Introduction

Timing of menarche, a landmark of the late stage of pubertal development, reflects the health status of a particular population, and in turn, has many potential health implications in later life. Early menarche is a well-known risk factor for breast cancer [1], [2], type 2 diabetes mellitus [3], metabolic syndrome [4], cardiovascular disease, cardiovascular disease mortality and all-cause mortality [5].

The interactions between genetic and environmental factors influence the age at which menarche occurs. It is estimated that approximately half of the variations in the timing of menarche can be ascribed by genetic influences [6]. Thus, identifying the modifiable factors affecting the timing of menarche is essential to reduce the risk of the chronic outcomes in later life. It seems that nutrition plays a pivotal role in determination the onset of menarche. Higher pre-pubertal body weight, body mass index (BMI), body fat, and height are linked to earlier age of menarche [7]–[9].

It has also been proposed that nutrition can influence timing of puberty beyond its effects on body composition [10]. Prospective observational studies suggest that higher intakes of fiber [11], vegetable protein [12], [13], animal fat [11], saturated fatty acid (SFA) [14] and mono-unsaturated fatty acid (MUFA) [11] are associated with later age at menarche. While, higher intakes of total fat[12], [14], poly-unsaturated fatty acid (PUFA) [15] and animal protein [15], [16] are related to higher risk of early menarche. Previous studies suggest that the consumption of cow’s milk increases the serum concentrations of insulin like-growth factor 1 (IGF-1) [17], [18] and promotes height growth in pre-pubertal ages [19], [20] but it is not clear whether milk intake influences pubertal development, as well. The data of a few studies available on the association between milk or total dairy intakes and menarche provide inconsistent findings [15], [17], [21]–[23]. Moreover, the individual effect of dairy products, other than milk, on age at menarche remains to be investigated. Dairy products are a complex group of foods that their physicochemical properties depend on their processing. As previous findings suggest that dairy products have not the same associations on height growth [19], [20], it is possible for them to have different relationships with menarche. Studies available are also less clear regarding the relation between Calcium (Ca), magnesium (Mg) and other nutrient intakes and age of menarche [14]–[16]. While no association with Ca intake throughout childhood and menarcheal age was observed in some prospective observational studies [14], [15], a randomized controlled trial showed a significant negative correlation between Ca intake during the intervention and age at menarche in pre-pubertal girls[24]. Considering the aforementioned, we examined the effects of pre-pubertal intakes of dairy products, including milk, yoghurt and cheese, on age at menarche using prospectively collected data from the Tehran Glucose and Lipid Study (TLGS). The influence of some dairy-related nutrients including Ca, Mg, and phosphorus (P) intakes on menarche were also evaluated separately.

## Methods

### Participants

The participants of the present study were selected from the Tehran Lipid and Glucose Study (TLGS), an ongoing prospective urban population-based study being conducted to identify the risk factors for non-communicable diseases in a representative sample of residents of Tehran, the capital of Iran. The rationale, sampling and data-collection procedures have been described elsewhere in detail [25]. In brief, the TLGS was initiated in 1999 with a random sample of 15005 participants aged ≥ 3 years enrolled from district No.13 of Tehran, and follow-up examinations have been conducted every 3 years thereafter, consisting of a face-to-face interview, physical and nutritional assessments, and clinical examinations, at the TLGS unit by trained specialists. Age distribution and socioeconomic status of the participants in the TLGS is representative of overall population of Tehran. Of the total participants recruited at baseline, dietary data were collected randomly for 1474 individuals, 190 of whom were girls aged ≥ 4 and ≤ 12 years. This study was conducted on 134 pre-pubertal healthy girls who were followed until reaching menarche with a median follow-up of 6.5 years. At beginning of the study, parents of each participant provided written informed consent, following approval by the ethics committee of the Research Institute for Endocrine Sciences of Shahid Beheshti University of Medical Sciences. This study was conducted in accordance with the principles of the Declaration of Helsinki.

### Data collection

Dietary intake at baseline, including dairy products and selected nutrients intake, was assessed by expert dietitians using two non-consecutive 24-h dietary recalls (24-hDR); the two week-days were selected randomly approximately 10 days apart. The first recall was completed at subject’s home and the second at the TLGS unit, both by the same dietitian. The household portions were converted to grams and the daily nutrients intakes were determined using nutritionist ΙΙΙ software (N-Squared Computing, Salem, OR, USA), which was modified for the Iranian food composition table. For this analysis, all types of fluid milk (as plain or flavored milk) and cheese (including feta cheese or traditional cheese) were aggregated to determine total milk and cheese intake. In the current study, Ca, Mg, and P intakes represent the total intakes of the mineral from all dietary sources.

During the follow-up, each participant was asked about the initiation of menarche and the age at which her first menstrual period occurred. The age at menarche was documented in completed whole years by asking an open question “At what age did you have your first menstrual period?”, and was confirmed with the probing question “Do you remember what grade you were when your menstruation began?” for girls and “How many years after your first menstruation did you get married?” for mothers.

Weight and height were measured according to standard protocols, using digital scales and tape measures respectively. BMI was calculated as weight (Kg) divided by height square (m^2^). Data on family size and smoking exposure were obtained using a standard, validated questionnaire.

### Statistical analysis

The age at menarche was analyzed as a dichotomous outcome, ≤ 12 and > 12 years. These categories are based on the mean age at menarche of Tehranian girls in TLGS [26] minus one standard deviation (SD). Characteristics of the participants were compared between two groups using Mann-Whitney U-test and independent-sample T-test for non-normally and normally distributed continuous variables, respectively and Chi-square test for categorical variables. For BMI and height, age-independent Z-scores were calculated using the 2007 WHO reference data. Underweight and overweight were defined as BMI-for-age Z-score below two standard deviations (SD) and above 1 SD of the reference data, respectively [27]. Nutritional comparisons between the two groups were made after determining energy intake per cm of height and dairy or other nutrients intake per 1000 Kcal of energy intake.

Age-adjusted dairy products and mineral intakes were analyzed as categorical variables, tertile or median, depending on the shape of their distribution and the proportion of non-users. Age-adjusted dairy products and nutrient intakes were computed as residuals from the regression model, with age as the independent variable and dietary intakes as the dependent variable. Odds ratios (ORs) and 95% CIs for age at menarche ≤ 12 years across the dairy products and nutrient intake categories were estimated by logistic regression after accounting for potential available covariates, using the following modeling strategy. In model 1, we adjusted for baseline energy and protein intake, interval between the age at study initiation and the age of menarche, and maternal age at menarche. Model 2 was adjusted additionally for baseline BMI Z-score. Model 3 was further adjusted for baseline height Z-score. These covariates were included in the analyses in accordance to previous studies that evaluated the association of dietary intake with age at menarche [11], [15], [23], [28]. Statistical analyses were performed using SPSS version 15 (SPSS Inc., Chicago, IL). A two-tailed P -value ≤ 0.05 was considered statistically significant.

## Results

The mean age at baseline of the 134 girls was 8.9 ± 2.4 years (mean ± SD) and median length of follow-up was 6.5 years with a range of 2.5–12 years. The mean age at menarche in our participants was 12.7 ± 1.3 years with a range of 9–18 years and median of 13.0 years. The prevalence of underweight and overweight were 11.3 and 9.8%, respectively. The baseline characteristics of participants at baseline according to age at menarche are presented in Table 1. There were no significant differences between girls who reached menarche at age ≤ 12 and those > 12 years regarding anthropometric variables, maternal age at menarche, family sizes and smoking exposure. As shown in the table, girls with age at menarche ≤ 12 years had significantly higher intakes of milk (P =  0.02), Mg (P =  0.04) and P (P =  0.01), and lower intake of cheese (P =  0.05) per 1000 Kcal energy than girls with age at menarche > 12 years.

**Table 1:** Participant characteristics according to the age of menarche.^1^

Variables	Menarche ≤ 12 years		p value
	Yes (n = 60)	No(n = 74)	
Age at baseline (years)	8 (6, 10)	10 (8, 11)	0.001
Interval between the age at study initiation and the age of menarche (years)	3 (1, 6)	4 ( 2, 6)	0.08
Height for age at baseline: Z score	–0.23 (–0.94, 0.40)^2^	–0.46 (–1.24, 0.47)	0.1
BMI for age at baseline: Z score	–0.49 (–0.95, 0.11)	–0.51 (–1.05, 0.27)	0.9
Weight status; No. (%)			
Underweight	6(10)	9(12.3)	
Overweight /obese	4(6.7)	9(12.3)	0.5
Maternal age at menarche(years)	13(13, 14)	13(13, 14)	0.1
Family size			
≤4	36 (60)	38 (51.4)	
5	15 (25)	14 (18.9%)	
≥6	9 (15)	22 (29.7)	0.1
Smoking exposure; No. (%)			
Yes	9 (15)	16 (21.6)	
No	51(85)	58 (78.4)	0.3
Energy intake (kcal/height)	14.5 (12.4, 16.7)	14.4 (12.3, 17.3)	0.6
Carbohydrate (% energy)	56.8±6.0^3^	58.6±7.2	0.1
Total fat (% energy)	33.6±6.1	32 ± 7.4	0.1
Protein (% energy)	11.3 ±2.2	11.2 ± 1.9	0.8
Milk intake (g/ 1000 kcal)^4^	51.2 (0.0, 113.2)	0.0 (0.0, 72.5)	0.02
Yoghurt intake (g/1000 kcal)	18.2 (0.0, 40.0)	24.7 (0.0, 54.9)	0.4
Cheese intake (g/ 1000 kcal)	3.1 (0.0, 5.9)	4.6 (1.5, 8.1)	0.05
Calcium intake (mg/1000 kcal)	295.9 (208.3, 402.2)	271.4 (234.3, 336.7)	0.3
Magnesium intake (mg/1000 kcal)	57.5 (44.2, 75.3)	49.8 (42.0, 63.7)	0.04
Phosphorus intake (mg/ 1000 kcal)	375.4 (290.8, 462.0)	326.4 (275.5, 379.4)	0.01

1 T-test and Mann-Whitney U-test was used for normally and non-normally distributed continuous variables, respectively, and chi-square test for categorical variables.

2 Median (interquartile range; IQR) (all such values).

3 Mean±SD (all such values).

4 n 16 values were missed for milk, yoghurt, and cheese intake.

As shown in Table 2, an increased risk of age at menarche ≤ 12 years was observed in girls who consumed higher amount of milk (> 34 g/day) compared to those with lower intake (≤ 34 g/day), after controlling for energy and protein intake at baseline, interval between the age at study initiation and the age of menarche, and maternal age at menarche (Model 1; OR: 2.28, p  =  0.04). Those in the middle tertile of cheese intake had a lower risk of reaching menarche ≤ 12 years (OR: 0.37, p  =  0.04) than those in the lowest tertile after controlling for energy and protein intake, interval between the age at study initiation and the age of menarche, and maternal age at menarche and there was no differences in risk between the highest and lowest tertiles. There were no significant associations between yoghurt and menarcheal age. In addition, girls whose intakes of Ca, Mg and P were higher had an increased risk of age at menarche ≤ 12 years, after controlling for energy and protein intake, interval between the age at study initiation and the age of menarche, and maternal age at menarche. These results remained significant after controlling for BMI Z-score at baseline (Model 2). After additional adjustment for height Z-score at baseline, the ORs for milk and cheese intake became statistically non-significant, whereas the associations between nutrient intakes and age at menarche remained significant (Model 3).

**Table 2:** Prospective associations between dairy, Ca, Mg, and P intakes and the risk of reaching menarche ≤ 12 years from logistic regression.^1^

Variables	n	Model 1^3^ OR (95%CI)	p	Model 2^4^ OR (95%CI)	p	Model 3^5^ OR (95%CI)	p
Milk (g/day)^2^							
≤ 34	62	1.00	0.04	1.00	0.04	1.00	0.08
>34	56	2.28 (1.03–5.05)		2.34 (1.05–5.18)		2.01 (0.93–4.76)	
Yogurt (g/day)^2^							
0	39	1.00		1.00		1.00	
< 80	40	1.14 (0.44–2.92)	0.8	1.06 (0.41–2.75)	0.9	0.95 (0.37–2.38)	0.6
≥ 80	39	0.80 (0.30–2.09)	0.6	0.75 (0.28–1.99)	0.5	0.72 (0.27–1.91)	0.7
Cheese (g/day)^2^							
0	39	1.00		1.00		1.00	
≤ 12.5	40	0.37 (0.14–0.96)	0.04	0.35 (0.13–0.92)	0.03	0.39 (0.15–1.05)	0.06
>12.5	39	0.54 (0.21–1.32)	0.2	0.48 (0.18–1.24)	0.1	0.54 (0.20–1.46)	0.2
Calcium (mg/day)							
≤530	67	1.00	0.006	1.00	0.009	1.00	0.01
>530	67	3.20 (1.39–7.42)		3.10(1.33–7.20)		3.03 (1.30–7.10)	
Magnesium (mg/day)							
≤ 106	68	1.00	0.02	1.00	0.03	1.00	0.02
> 106	66	2.43 (1.12–5.27)		2.42 (1.11–5.29)		2.59 (1.17–5.77)	
Phosphorus(mg/day)							
≤ 647	67	1.00	0.005	1.00	0.005	1.00	0.005
> 647	67	3.37 (1.44–7.87)		3.43 (1.47–8.05)		3.43 (1.45–8.13)	

1 Dietary intake was adjusted for age at baseline by residuals.

2 n 16 values were missed for milk, yoghurt, and cheese intake.

3 Model 1 adjusted for energy and protein intake at baseline, interval between the age at study initiation and the age of menarche, and maternal age at menarche.

4 Model 2 adjusted for variables in model 1 and BMI Z-score at baseline.

5 Model 3 adjusted for variables in model 2 and height Z-score at baseline.

## Discussion

Our prospective study suggests that girls with higher intake of milk, experience menarcheal age earlier compared to those with a lower intake. This association was observed independently of energy, protein and BMI Z-score at baseline. Controlling for height Z-score at baseline made the association non-significant, suggesting that height may have mediating effect on the association between milk intake and age at menarche.

Few studies have been conducted to examine the association between milk intake and age at menarche [17], [21]-[23], [29]. In accordance to our finding, data of girls aged 9-12 years, from NHANES 1994-2004 showed that individuals in the middle tertile of milk intake, < 244 g/day, had a lower risk of early menarche than did those in the highest tertile (> 245 g/day). In addition, girls with intakes between 100-229 Kcal/day from dairy had higher risk of early menarche than those with intakes > 230 Kcal/day after controlling for height and overweight status [23]. In a recently published study on a representative Hong Kong Chinese birth cohort, cow’s milk consumption was not associated with age at pubertal onset in children aged 7-14 years [29]; however, in the study, the effect of milk was evaluated as frequency of milk intake and total energy intake was not controlled for. This discrepancy in findings from observational studies may be due to variation in study design or extent of adjustment for potentially confounding factors. Milk supplements in pre-menarche girl, aged 10–12 years, showed no significant effect on age at menarche [17], [21], [22]. Since it has been proposed that diet in early to mid-childhood is more strongly related to the occurrence of menarche than diet later in childhood [15], it is likely that milk supplementation at ages of close to time of menarche did not affect age of menarche in the trials. It is also possible that the amount of milk supplementation in these studies was not high enough to influence the age of menarche in interventions as compared to controls.

One of the possible mechanisms by which milk intake can accelerate growth and development, might be due, at least in part, to increased insulin-like growth factor-1(IGF-1) concentration. IGF-1 is an important mitogen peptide that can increase estrogen production through stimulating the adrenal androgen secretion [30], [31] or enhancing the secretion of gonadotropin-releasing hormone (GnRH) by hypothalamic neurons [10]. Cow’s milk contains IGF-1, which can be enhanced by administrating of recombinant growth hormone to cows in order to increase milk production [32]. In addition, several identified nutrients of milk including protein, Ca, Mg, and P or some yet unknown bioactive components can stimulate IGF-1 production [18], [33], [34].

In the present study, higher intakes Ca, Mg, and P were related consistently to the risk of earlier menarche. The findings suggest that Ca, Mg, and P are some components of milk, which might be involved in the menarche-promoting effect observed. Previous observational studies regarding the effect of nutrients including Ca and Mg provide inconsistent results [14], [15], [23], [24]. In a randomized controlled trial, girls who received 850 mg/day as milk-Ca-P extract from age 7.9–8.9 years, experienced menarche 5.4 months earlier than the placebo groups. Also, total calcium intake during the intervention was inversely correlated with menarcheal age, even after adjusting for baseline body weight and height [24]. Low calcium intake was associated with a decrease in all aromatizable adrenal androgens and an increase in sex hormones binding globulin (SHBG); leading to delayed pubertal development in girls, aged 11–14 years [31]. There is also some evidence that Mg and P are involved in modulation of anabolic hormones secretion including testosterone and IGF-1 in middle-aged and elderly [34], [35]. The relationship between Mg, P, and sex-steroid hormones in children and adolescents deserve investigation to ascertain their potential effects on puberty.

We have observed an opposite effect for cheese intake and risk of age at menarche ≤ 12 years as compared to milk intake. The lower risk of age at menarche ≤ 12 years in the middle tertile of cheese intake as compared to the lowest was significant in model 1 and 2. Furthermore, yogurt intake had no association with the risk of earlier age at menarche. Dairy products are a complex group of foods and the compositions can be changed based on their processing; for instance, previous findings suggest that IGF-1 in milk is less influenced by homogenization and pasteurization than the processing of milk to dairy products [36]. Therefore, it is possible that IGF-1 or substances stimulating the endogenous production of IGF-1 or even unknown components in milk, responsible for promoting age at menarche, be inactivated during processing [37].

Attaining age at menarche earlier has diverse impacts on health [28]; earlier age of menarche increases the risk of breast cancer later in life[1], [2], on the other hand it is associated with higher bone mineral density and lower risk of osteoporosis [38]. Hence, before public health recommendations can be made, long-term health consequences of reaching menarche earlier in those with higher intakes of milk need to be elucidated.

A major strength of the current study is that we could prospectively investigate the associations between mid-childhood consumption of dairy and age of menarche. The information on maternal age at menarche obtained from the TLGS study is another strength of the study, which made it possible to control for potential genetic influences on menarche. To the best of our Knowledge, this is a first time that the effects of other dairy products including cheese and yoghurt on age at menarche were examined individually. Furthermore, we followed our participants long enough for all of them to reach menarche.

This study has some limitations. First, dietary intakes were used only at baseline; however, it has been shown that dietary intakes tend to track throughout childhood into adolescence [39], [40]. Therefore, we consider dietary intakes at baseline as a usual, established pattern of intake. Second, dietary intakes were determined by using 2-day 24-hour food recalls that may have assessed dairy and mineral intakes less precisely than if measured on 7- day 24-hour food recalls or if the FFQ were used; however, this may have underestimated the true effects so it is unlikely to be an explanation for the associations observed. Third, socioeconomic status (SES) as a potential confounding factor was not taken into account. In Iran, age at menarche is related to SES [41]. It is not clear whether milk and dairy intakes are also socially patterned but high cost of healthy food and inadequate knowledge/information were recognized as barriers of choosing healthy nutrition in Iran [42]. Based on the above evidence and due to lack of valid data on SES in the present study, our findings might be confounded by socioeconomic status. Fourth, we could not separate high and low fat dairy products and hence did not differentiate the effects of high and low fat dairy products, whereas previous studies suggest that these may influence age of menarche differently [11], [23]. Finally, high consumption of milk and nutrient intakes may reflect overall better dietary quality and healthier life style in those with early menarche. Thus, the associations observed may be attributed to the other characteristics related to the timing of menarche. However, we cannot rule out such residual confounding.

In summary, findings of this community based study cohort provided evidence that milk intake in pre-puberty might hasten the timing of menarche. It seems that dairy products including milk, cheese and yoghurt may have different effects on puberty. Higher intakes of Ca, Mg, and P were also related to an earlier age of menarche. Further studies are needed to confirm these findings, and to clarify the possible effects of different types of milk and dairy products individually, regarding to their content of fat or technological processing. Investigating the relationship between dairy intakes and other pubertal events are also recommended in future studies.
